# μ-Oxido-bis­[bis­(penta­fluoro­phenolato)(η^5^-penta­methyl­cyclo­penta­dien­yl)titanium(IV)]

**DOI:** 10.1107/S1600536811029357

**Published:** 2011-07-30

**Authors:** Junseong Lee, Youngjo Kim

**Affiliations:** a Department of Chemistry, Chonnam National University, Gwangju 500-757, Republic of Korea; b Department of Chemistry, Chungbuk National University, Cheongju, Chungbuk 361-763, Republic of Korea

## Abstract

The dinuclear title complex, [Ti_2_(C_10_H_15_)_2_(C_6_F_5_O)_4_O], features two Ti^IV^ atoms bridged by an O atom, which lies on an inversion centre. The Ti^IV^ atom is bonded to a η^5^-penta­methyl­cyclo­penta­dienyl ring, two penta­fluoro­phenolate anions and to the bridging O atom. The environment around the Ti^IV^ atom can be considered as a distorted tetra­hedron. The cyclo­penta­dienyl ring is disordered over two sets of sites [site occupancy = 0.824 (8) for the major component].

## Related literature

For the related titanium complexes, Cp^*^Ti(OCH_2_C_6_F_5_)_3_ and Cp^*^Ti(OC_6_F_5_)_3_, see: Lee *et al.* (2007 ▶) and for [Ti_2_(η^5^-C_5_Me_5_)_2_(OCH_2_C_6_F_5_)_4_O], see: Lee & Kim (2011 ▶). For the use of dinuclear titanium complexes with a cyclo­penta­dienyl ligand in organometallic catalysis, see: Noh *et al.* (2006 ▶); Wu *et al.* (2007 ▶); Yoon *et al.* (2011 ▶). For the Ti—O—Ti angle in related structures, see: Gowik *et al.* (1990 ▶); Thewalt & Schomburg (1977 ▶); Wu *et al.* (2007 ▶).
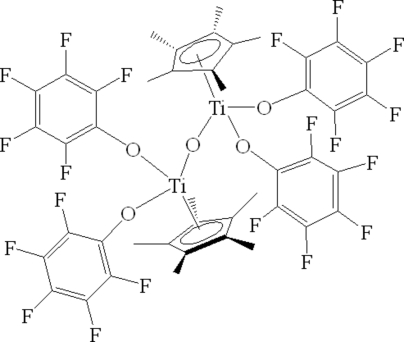


## Experimental

### 

#### Crystal data

[Ti_2_(C_10_H_15_)_2_(C_6_F_5_O)_4_O]
*M* *_r_* = 1114.48
Triclinic, 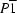

*a* = 8.7472 (17) Å
*b* = 11.823 (2) Å
*c* = 12.923 (3) Å
α = 112.00 (3)°
β = 109.24 (3)°
γ = 97.36 (3)°
*V* = 1120.6 (4) Å^3^
*Z* = 1
Mo *K*α radiation
μ = 0.49 mm^−1^
*T* = 293 K
0.12 × 0.10 × 0.08 mm

#### Data collection

Bruker SMART 1K CCD diffractometer
Absorption correction: multi-scan (*SADABS*; Bruker, 2004 ▶) *T*_min_ = 0.94, *T*_max_ = 0.96
12962 measured reflections
5069 independent reflections
3892 reflections with *I* > 2σ(*I*)
*R*_int_ = 0.033

#### Refinement

*R*[*F* ^2^ > 2σ(*F* ^2^)] = 0.049
*wR*(*F* ^2^) = 0.141
*S* = 1.03
5069 reflections
423 parameters
49 restraints
H-atom parameters constrained
Δρ_max_ = 0.28 e Å^−3^
Δρ_min_ = −0.34 e Å^−3^

### 

Data collection: *SMART* (Bruker, 2004 ▶); cell refinement: *SAINT* (Bruker, 2004 ▶); data reduction: *SAINT*; program(s) used to solve structure: *SHELXS97* (Sheldrick, 2008 ▶); program(s) used to refine structure: *SHELXL97* (Sheldrick, 2008 ▶); molecular graphics: *ORTEP-3 for Windows* (Farrugia, 1997 ▶); software used to prepare material for publication: *SHELXTL* (Sheldrick, 2008 ▶).

## Supplementary Material

Crystal structure: contains datablock(s) I, global. DOI: 10.1107/S1600536811029357/bx2364sup1.cif

Supplementary material file. DOI: 10.1107/S1600536811029357/bx2364Isup2.cdx

Structure factors: contains datablock(s) I. DOI: 10.1107/S1600536811029357/bx2364Isup3.hkl

Additional supplementary materials:  crystallographic information; 3D view; checkCIF report

## Figures and Tables

**Fig. 1.**
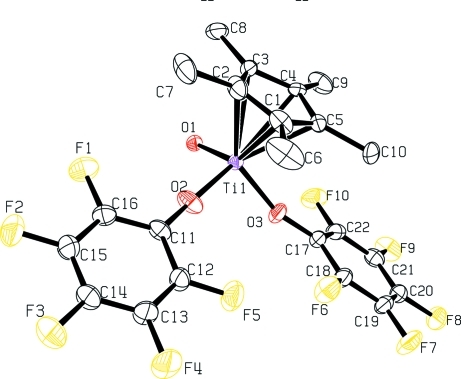
The asymmetric unit of the title compound (I), showing the atom-labelling scheme. Displacement ellipsoids are drawn at the 20% probability level. H atoms are omitted for clarity.

**Fig. 2.**
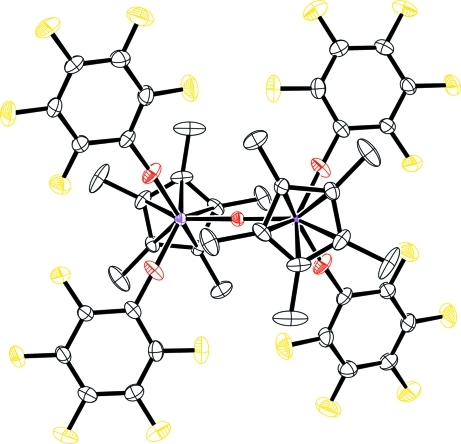
The molecular structure of the title compound (I). Displacement ellipsoids are drawn at the 20% probability level. H atoms are omitted for clarity.

## supplementary crystallographic information

### Comment

Dinuclear titanium complexes containing a cyclopentadienyl ligand have attracted considerable attention in the fields of organometallic catalysis (Noh *et al.*, 2006; Wu *et al.*, 2007; Yoon *et al.*, 2011). Recently, we have reported the facile synthesis of Cp^*^Ti(OCH_2_C_6_F_5_)_3_ and Cp^*^Ti(OC_6_F_5_)_3_ (Cp^*^ = η^5^-pentamethylcyclopentadienyl) (Lee *et al.*, 2007). We have also reported the X-ray structure of [Ti_2_(η^5^-C_5_Me_5_)_2_(OCH_2_C_6_F_5_)_4_O] (Lee & Kim 2011). In continuation of our systematic studies on bimetallic pentamethylcyclopentadienyltitanium derivative using previously synthesized Cp^*^Ti(OC_6_F_5_)_3_, the title complex (I) has been investigated.

The title compound (I) is the main product of the reaction of Cp^*^Ti(OC_6_F_5_)_3_ with water in dichloromethane solution. In (I) (Fig. 1), the dinuclear structure shows two Ti atoms bridged by an oxygen atom, which is lies on inversion centre, Fig. 2 , with approximately C_2_ symmetry. Ti atom bonded with bridging oxygen atom, a Cp ring and two pentafluorophenolate groups, having distorted tetrahedron geometry.

A disorder of Cp* rings was observed in a ratio of 0.824 (8) and 0.176 (8) for C1—C10 and C1A—C10A, respectively. The Ti—C and Ti—O distances are in the range of 2.337 (16) - 2.400 (11) Å and 1.8184 (11) - 1.854 (2) Å, respectively. The Ti—O—Ti angle is almost linear [180.00 (4) °], which falls within the observed range (154 - 180°) for the previous reported compounds (Wu *et al.*, 2007; Thewalt & Schomburg, 1977; Gowik *et al.*, 1990; Lee & Kim, 2011). Whereas Cp^*^ and phenyl rings are almost perpendicular in [Ti_2_(η^5^-C_5_Me_5_)_2_(OCH_2_C_6_F_5_)_4_O], the Cp^*^ ring and phenyl rings are almost parallel with the dihedral angles of 20.8 (6) ° and 10.2 (6) ° and there is π-π interaction between and Cp ring and phenyl ring (C17—C22) with the perpendicular distance of 3.396 Å.

### Experimental

Complex (I) was synthesized by the hydrolysis of Cp^*^Ti(OC_6_F_5_)_3_. The crystal was obtained by slow evaporation of methylene chloride as a solvent in refrigerator.

### Refinement

The disordered Cp* ring was modeled by splitting the atoms into two components (C1 - C10 and C1A—C10A), the site occupation factors of which refined in a ratio of 0.824 (8):0.176 (8). H atoms were positioned geometrically and refined using a riding model, with C—H distances fixed to 0.96 (methyl CH_3_), 0.97 (methylene CH_2_)and with *U*_iso_(H) = 1.2 (1.5 for methyl groups) times *U*_eq_(C).

### Crystal data

**Table:**

[Ti_2_(C_10_H_15_)_2_(C_6_F_5_O)_4_O]	*Z* = 1
*M**_r_* = 1114.48	*F*(000) = 558
Triclinic, *P*1	*D*_x_ = 1.651 Mg m^−^^3^
Hall symbol: -P 1	Mo *K*α radiation, λ = 0.71073 Å
*a* = 8.7472 (17) Å	Cell parameters from 5069 reflections
*b* = 11.823 (2) Å	θ = 1.9–28.3°
*c* = 12.923 (3) Å	µ = 0.49 mm^−^^1^
α = 112.00 (3)°	*T* = 293 K
β = 109.24 (3)°	Block, yellow
γ = 97.36 (3)°	0.12 × 0.10 × 0.08 mm
*V* = 1120.6 (4) Å^3^	

### Data collection

**Table:**

Bruker SMART 1K CCD diffractometer	5069 independent reflections
Radiation source: fine-focus sealed tube	3892 reflections with *I* > 2σ(*I*)
graphite	*R*_int_ = 0.033
profile data from /ω scans	θ_max_ = 28.3°, θ_min_ = 1.9°
Absorption correction: multi-scan (*SADABS*; Bruker, 2004)	*h* = −11→11
*T*_min_ = 0.94, *T*_max_ = 0.96	*k* = −15→15
12962 measured reflections	*l* = −17→16

### Refinement

**Table:**

Refinement on *F*^2^	Primary atom site location: structure-invariant direct methods
Least-squares matrix: full	Secondary atom site location: difference Fourier map
*R*[*F*^2^ > 2σ(*F*^2^)] = 0.049	Hydrogen site location: inferred from neighbouring sites
*wR*(*F*^2^) = 0.141	H-atom parameters constrained
*S* = 1.03	*w* = 1/[σ^2^(*F*_o_^2^) + (0.0747*P*)^2^ + 0.3233*P*] where *P* = (*F*_o_^2^ + 2*F*_c_^2^)/3
5069 reflections	(Δ/σ)_max_ < 0.001
423 parameters	Δρ_max_ = 0.28 e Å^−^^3^
49 restraints	Δρ_min_ = −0.34 e Å^−^^3^

### Special details

**Table:**

Geometry. All e.s.d.'s (except the e.s.d. in the dihedral angle between two l.s. planes) are estimated using the full covariance matrix. The cell e.s.d.'s are taken into account individually in the estimation of e.s.d.'s in distances, angles and torsion angles; correlations between e.s.d.'s in cell parameters are only used when they are defined by crystal symmetry. An approximate (isotropic) treatment of cell e.s.d.'s is used for estimating e.s.d.'s involving l.s. planes.
Refinement. Refinement of *F*^2^ against ALL reflections. The weighted *R*-factor *wR* and goodness of fit *S* are based on *F*^2^, conventional *R*-factors *R* are based on *F*, with *F* set to zero for negative *F*^2^. The threshold expression of *F*^2^ > σ(*F*^2^) is used only for calculating *R*-factors(gt) *etc*. and is not relevant to the choice of reflections for refinement. *R*-factors based on *F*^2^ are statistically about twice as large as those based on *F*, and *R*- factors based on ALL data will be even larger.

### Fractional atomic coordinates and isotropic or equivalent isotropic displacement parameters (Å^2^)

**Table:**

	*x*	*y*	*z*	*U*_iso_*/*U*_eq_	Occ. (<1)
Ti1	0.09529 (5)	0.15447 (4)	0.13341 (3)	0.04162 (14)	
O1	0.0000	0.0000	0.0000	0.0441 (5)	
O2	0.1777 (3)	0.1137 (2)	0.26188 (17)	0.0781 (6)	
O3	0.2933 (2)	0.22063 (19)	0.1261 (2)	0.0754 (6)	
C1	0.0139 (11)	0.3210 (8)	0.2536 (4)	0.0689 (19)	0.824 (8)
C2	−0.1240 (12)	0.2120 (9)	0.1862 (9)	0.068 (2)	0.824 (8)
C3	−0.1767 (11)	0.1821 (8)	0.0620 (7)	0.0561 (16)	0.824 (8)
C4	−0.0668 (9)	0.2698 (6)	0.0523 (5)	0.0511 (10)	0.824 (8)
C5	0.0508 (7)	0.3587 (4)	0.1715 (7)	0.0576 (13)	0.824 (8)
C6	0.1057 (11)	0.3864 (8)	0.3922 (4)	0.143 (4)	0.824 (8)
H6A	0.0514	0.4471	0.4261	0.215*	0.824 (8)
H6B	0.2211	0.4294	0.4154	0.215*	0.824 (8)
H6C	0.1028	0.3239	0.4228	0.215*	0.824 (8)
C7	−0.2140 (9)	0.1381 (8)	0.2349 (8)	0.125 (3)	0.824 (8)
H7A	−0.1457	0.1647	0.3198	0.188*	0.824 (8)
H7B	−0.2318	0.0486	0.1894	0.188*	0.824 (8)
H7C	−0.3211	0.1547	0.2260	0.188*	0.824 (8)
C8	−0.3250 (6)	0.0759 (5)	−0.0432 (6)	0.095 (2)	0.824 (8)
H8A	−0.4250	0.1038	−0.0549	0.142*	0.824 (8)
H8B	−0.3400	0.0040	−0.0259	0.142*	0.824 (8)
H8C	−0.3049	0.0518	−0.1162	0.142*	0.824 (8)
C9	−0.0767 (9)	0.2720 (7)	−0.0655 (5)	0.0956 (19)	0.824 (8)
H9A	−0.1191	0.1865	−0.1298	0.143*	0.824 (8)
H9B	0.0339	0.3104	−0.0556	0.143*	0.824 (8)
H9C	−0.1513	0.3205	−0.0862	0.143*	0.824 (8)
C10	0.1810 (6)	0.4768 (4)	0.2046 (8)	0.118 (3)	0.824 (8)
H10A	0.1343	0.5475	0.2204	0.177*	0.824 (8)
H10B	0.2136	0.4645	0.1380	0.177*	0.824 (8)
H10C	0.2782	0.4940	0.2765	0.177*	0.824 (8)
C1A	−0.022 (4)	0.282 (3)	0.2591 (15)	0.043 (5)	0.176 (8)
C2A	−0.155 (4)	0.189 (2)	0.1539 (19)	0.044 (9)	0.176 (8)
C3A	−0.148 (4)	0.201 (3)	0.0513 (14)	0.050 (11)	0.176 (8)
C4A	−0.016 (3)	0.309 (2)	0.094 (2)	0.037 (5)	0.176 (8)
C5A	0.061 (2)	0.3606 (17)	0.224 (2)	0.047 (5)	0.176 (8)
C6A	−0.006 (4)	0.304 (3)	0.3866 (18)	0.105 (8)	0.176 (8)
H6A1	−0.0817	0.3510	0.4074	0.158*	0.176 (8)
H6A2	0.1083	0.3520	0.4447	0.158*	0.176 (8)
H6A3	−0.0332	0.2238	0.3882	0.158*	0.176 (8)
C7A	−0.288 (3)	0.080 (2)	0.139 (3)	0.092 (7)	0.176 (8)
H7A1	−0.2506	0.0669	0.2111	0.138*	0.176 (8)
H7A2	−0.3035	0.0036	0.0693	0.138*	0.176 (8)
H7A3	−0.3929	0.1007	0.1267	0.138*	0.176 (8)
C8A	−0.278 (3)	0.122 (2)	−0.0793 (15)	0.077 (7)	0.176 (8)
H8A1	−0.2424	0.1456	−0.1331	0.115*	0.176 (8)
H8A2	−0.3850	0.1380	−0.0856	0.115*	0.176 (8)
H8A3	−0.2894	0.0335	−0.1019	0.115*	0.176 (8)
C9A	0.021 (3)	0.357 (2)	0.010 (2)	0.076 (7)	0.176 (8)
H9A1	0.0339	0.2895	−0.0541	0.114*	0.176 (8)
H9A2	0.1237	0.4262	0.0551	0.114*	0.176 (8)
H9A3	−0.0704	0.3866	−0.0260	0.114*	0.176 (8)
C10A	0.204 (3)	0.4821 (17)	0.310 (2)	0.092 (7)	0.176 (8)
H10D	0.1610	0.5542	0.3185	0.138*	0.176 (8)
H10E	0.2883	0.4859	0.2784	0.138*	0.176 (8)
H10F	0.2537	0.4832	0.3892	0.138*	0.176 (8)
C11	0.2320 (4)	0.0988 (3)	0.3627 (2)	0.0592 (7)	
C12	0.3854 (4)	0.1761 (3)	0.4611 (3)	0.0705 (8)	
C13	0.4441 (4)	0.1573 (3)	0.5639 (3)	0.0803 (9)	
C14	0.3539 (5)	0.0620 (4)	0.5728 (3)	0.0799 (9)	
C15	0.2040 (5)	−0.0145 (3)	0.4798 (3)	0.0792 (9)	
C16	0.1435 (4)	0.0036 (3)	0.3765 (3)	0.0714 (8)	
C17	0.4133 (3)	0.2970 (2)	0.1221 (3)	0.0570 (6)	
C18	0.5478 (3)	0.3887 (3)	0.2276 (3)	0.0613 (7)	
C19	0.6739 (3)	0.4667 (3)	0.2236 (3)	0.0653 (7)	
C20	0.6712 (3)	0.4546 (3)	0.1139 (3)	0.0641 (7)	
C21	0.5425 (4)	0.3650 (3)	0.0082 (3)	0.0639 (7)	
C22	0.4165 (3)	0.2878 (3)	0.0131 (3)	0.0627 (7)	
F1	−0.0057 (3)	−0.0725 (2)	0.2872 (2)	0.1189 (8)	
F2	0.1130 (3)	−0.1077 (3)	0.4882 (3)	0.1323 (10)	
F3	0.4136 (3)	0.0438 (3)	0.6738 (2)	0.1240 (9)	
F4	0.5933 (3)	0.2317 (3)	0.6553 (2)	0.1361 (11)	
F5	0.4779 (3)	0.2697 (2)	0.4549 (2)	0.1213 (9)	
F6	0.5545 (3)	0.4040 (2)	0.33688 (18)	0.0986 (7)	
F7	0.7987 (3)	0.5554 (2)	0.32698 (19)	0.1061 (7)	
F8	0.7934 (3)	0.5323 (2)	0.1102 (2)	0.0969 (7)	
F9	0.5402 (3)	0.3521 (2)	−0.09978 (19)	0.1006 (7)	
F10	0.2916 (2)	0.20016 (19)	−0.09189 (18)	0.0996 (7)	

### Atomic displacement parameters (Å^2^)

**Table:**

	*U*^11^	*U*^22^	*U*^33^	*U*^12^	*U*^13^	*U*^23^
Ti1	0.0396 (2)	0.0401 (2)	0.0364 (2)	0.00442 (16)	0.00860 (16)	0.01646 (17)
O1	0.0409 (11)	0.0429 (11)	0.0414 (11)	0.0072 (9)	0.0107 (9)	0.0186 (9)
O2	0.0982 (16)	0.0952 (16)	0.0437 (10)	0.0458 (13)	0.0178 (10)	0.0378 (11)
O3	0.0496 (10)	0.0548 (11)	0.1166 (17)	0.0041 (9)	0.0344 (11)	0.0360 (12)
C1	0.094 (6)	0.067 (5)	0.042 (2)	0.040 (4)	0.025 (2)	0.018 (2)
C2	0.079 (6)	0.082 (6)	0.087 (4)	0.039 (4)	0.054 (4)	0.059 (4)
C3	0.042 (2)	0.048 (2)	0.074 (4)	0.0132 (17)	0.023 (3)	0.024 (3)
C4	0.051 (3)	0.054 (3)	0.050 (3)	0.018 (2)	0.018 (2)	0.026 (2)
C5	0.052 (2)	0.039 (2)	0.072 (4)	0.0096 (17)	0.016 (3)	0.023 (3)
C6	0.190 (7)	0.153 (6)	0.040 (2)	0.105 (6)	0.020 (3)	0.002 (3)
C7	0.142 (6)	0.171 (7)	0.207 (8)	0.100 (5)	0.139 (6)	0.154 (7)
C8	0.047 (2)	0.066 (3)	0.124 (5)	0.016 (2)	0.005 (2)	0.020 (3)
C9	0.113 (4)	0.146 (6)	0.083 (3)	0.076 (4)	0.056 (3)	0.081 (4)
C10	0.068 (3)	0.048 (2)	0.217 (8)	0.011 (2)	0.042 (4)	0.054 (3)
C1A	0.046 (12)	0.045 (14)	0.033 (9)	0.014 (9)	0.006 (8)	0.023 (9)
C2A	0.019 (6)	0.014 (6)	0.064 (18)	−0.007 (5)	0.005 (8)	−0.005 (8)
C3A	0.039 (17)	0.08 (3)	0.016 (7)	0.021 (15)	0.005 (8)	0.009 (9)
C4A	0.035 (8)	0.040 (9)	0.040 (9)	0.010 (6)	0.012 (7)	0.024 (7)
C5A	0.045 (9)	0.038 (8)	0.049 (14)	−0.002 (6)	0.006 (10)	0.026 (10)
C6A	0.123 (12)	0.118 (12)	0.082 (10)	0.042 (9)	0.046 (8)	0.046 (8)
C7A	0.091 (10)	0.086 (10)	0.113 (11)	0.024 (7)	0.053 (8)	0.048 (8)
C8A	0.065 (13)	0.069 (14)	0.045 (9)	0.032 (11)	−0.010 (8)	−0.002 (8)
C9A	0.097 (16)	0.102 (17)	0.086 (16)	0.058 (14)	0.062 (14)	0.070 (14)
C10A	0.086 (10)	0.068 (9)	0.103 (11)	0.014 (7)	0.034 (8)	0.025 (7)
C11	0.0689 (17)	0.0690 (17)	0.0382 (12)	0.0267 (14)	0.0155 (12)	0.0260 (12)
C12	0.0753 (19)	0.0699 (18)	0.0567 (16)	0.0061 (15)	0.0133 (14)	0.0352 (15)
C13	0.076 (2)	0.089 (2)	0.0467 (15)	−0.0008 (17)	−0.0011 (14)	0.0308 (15)
C14	0.090 (2)	0.098 (2)	0.0553 (17)	0.0231 (19)	0.0188 (16)	0.0486 (18)
C15	0.081 (2)	0.087 (2)	0.079 (2)	0.0182 (18)	0.0290 (18)	0.0514 (19)
C16	0.0598 (17)	0.0742 (19)	0.0582 (17)	0.0108 (15)	0.0066 (14)	0.0254 (15)
C17	0.0430 (13)	0.0470 (13)	0.0771 (18)	0.0104 (11)	0.0218 (12)	0.0270 (13)
C18	0.0543 (15)	0.0652 (16)	0.0599 (16)	0.0041 (12)	0.0187 (12)	0.0315 (14)
C19	0.0480 (14)	0.0640 (17)	0.0682 (17)	−0.0019 (12)	0.0115 (13)	0.0301 (14)
C20	0.0533 (15)	0.0662 (17)	0.087 (2)	0.0161 (13)	0.0329 (15)	0.0448 (16)
C21	0.0700 (18)	0.0759 (19)	0.0647 (17)	0.0365 (16)	0.0361 (15)	0.0379 (15)
C22	0.0483 (14)	0.0564 (15)	0.0632 (17)	0.0187 (12)	0.0132 (12)	0.0138 (13)
F1	0.0778 (14)	0.1114 (18)	0.0993 (16)	−0.0091 (12)	−0.0151 (12)	0.0346 (14)
F2	0.1154 (19)	0.140 (2)	0.156 (2)	0.0004 (17)	0.0436 (18)	0.102 (2)
F3	0.141 (2)	0.163 (2)	0.0812 (14)	0.0342 (18)	0.0237 (14)	0.0898 (16)
F4	0.1177 (19)	0.133 (2)	0.0750 (13)	−0.0343 (15)	−0.0313 (13)	0.0469 (14)
F5	0.1201 (19)	0.1158 (18)	0.1085 (17)	−0.0161 (14)	0.0138 (14)	0.0749 (15)
F6	0.0966 (14)	0.1208 (17)	0.0711 (12)	−0.0025 (12)	0.0276 (11)	0.0520 (12)
F7	0.0747 (12)	0.1052 (16)	0.0829 (13)	−0.0316 (11)	−0.0004 (10)	0.0330 (12)
F8	0.0789 (12)	0.1069 (15)	0.1440 (19)	0.0200 (11)	0.0626 (13)	0.0825 (15)
F9	0.1248 (17)	0.1330 (19)	0.0800 (13)	0.0640 (15)	0.0616 (13)	0.0580 (13)
F10	0.0745 (12)	0.0846 (13)	0.0749 (12)	0.0138 (10)	0.0026 (10)	−0.0024 (10)

### Geometric parameters (Å, °)

**Table:**

Ti1—O1	1.8184 (11)	C2A—C7A	1.524 (16)
Ti1—O2	1.8464 (19)	C3A—C4A	1.395 (16)
Ti1—O3	1.854 (2)	C3A—C8A	1.516 (14)
Ti1—C3A	2.28 (4)	C4A—C5A	1.421 (15)
Ti1—C4A	2.320 (19)	C4A—C9A	1.510 (14)
Ti1—C2	2.336 (9)	C5A—C10A	1.515 (15)
Ti1—C4	2.356 (5)	C6A—H6A1	0.9600
Ti1—C2A	2.36 (4)	C6A—H6A2	0.9600
Ti1—C1	2.365 (7)	C6A—H6A3	0.9600
Ti1—C3	2.366 (10)	C7A—H7A1	0.9600
Ti1—C1A	2.37 (3)	C7A—H7A2	0.9600
Ti1—C5A	2.38 (2)	C7A—H7A3	0.9600
O1—Ti1^i^	1.8184 (11)	C8A—H8A1	0.9600
O2—C11	1.318 (3)	C8A—H8A2	0.9600
O3—C17	1.321 (3)	C8A—H8A3	0.9600
C1—C2	1.394 (8)	C9A—H9A1	0.9600
C1—C5	1.404 (7)	C9A—H9A2	0.9600
C1—C6	1.518 (6)	C9A—H9A3	0.9600
C2—C3	1.400 (7)	C10A—H10D	0.9600
C2—C7	1.529 (6)	C10A—H10E	0.9600
C3—C4	1.391 (7)	C10A—H10F	0.9600
C3—C8	1.500 (7)	C11—C16	1.384 (4)
C4—C5	1.415 (6)	C11—C12	1.394 (4)
C4—C9	1.506 (5)	C12—F5	1.329 (3)
C5—C10	1.501 (6)	C12—C13	1.370 (4)
C6—H6A	0.9600	C13—F4	1.335 (4)
C6—H6B	0.9600	C13—C14	1.355 (5)
C6—H6C	0.9600	C14—F3	1.347 (3)
C7—H7A	0.9600	C14—C15	1.350 (5)
C7—H7B	0.9600	C15—F2	1.335 (4)
C7—H7C	0.9600	C15—C16	1.373 (4)
C8—H8A	0.9600	C16—F1	1.332 (3)
C8—H8B	0.9600	C17—C22	1.382 (4)
C8—H8C	0.9600	C17—C18	1.389 (4)
C9—H9A	0.9600	C18—F6	1.335 (3)
C9—H9B	0.9600	C18—C19	1.372 (4)
C9—H9C	0.9600	C19—F7	1.331 (3)
C10—H10A	0.9600	C19—C20	1.362 (4)
C10—H10B	0.9600	C20—F8	1.343 (3)
C10—H10C	0.9600	C20—C21	1.361 (4)
C1A—C2A	1.385 (16)	C21—F9	1.340 (3)
C1A—C5A	1.403 (16)	C21—C22	1.371 (4)
C1A—C6A	1.521 (16)	C22—F10	1.337 (3)
C2A—C3A	1.405 (16)		
			
O1—Ti1—O2	103.35 (8)	C10—C5—Ti1	125.5 (4)
O1—Ti1—O3	104.10 (8)	C2A—C1A—C5A	107.1 (13)
O2—Ti1—O3	101.46 (11)	C2A—C1A—C6A	122 (2)
O1—Ti1—C3A	86.0 (7)	C5A—C1A—C6A	129 (2)
O2—Ti1—C3A	132.7 (6)	C2A—C1A—Ti1	72.5 (19)
O3—Ti1—C3A	121.3 (6)	C5A—C1A—Ti1	73.3 (16)
O1—Ti1—C4A	111.5 (6)	C6A—C1A—Ti1	130 (2)
O2—Ti1—C4A	139.0 (5)	C1A—C2A—C3A	109.5 (13)
O3—Ti1—C4A	90.6 (4)	C1A—C2A—C7A	129.5 (19)
C3A—Ti1—C4A	35.3 (5)	C3A—C2A—C7A	120.9 (18)
O1—Ti1—C2	106.3 (3)	C1A—C2A—Ti1	73.4 (19)
O2—Ti1—C2	92.3 (2)	C3A—C2A—Ti1	69 (2)
O3—Ti1—C2	142.6 (2)	C7A—C2A—Ti1	120 (2)
C3A—Ti1—C2	41.5 (6)	C4A—C3A—C2A	107.4 (12)
C4A—Ti1—C2	58.1 (4)	C4A—C3A—C8A	126.7 (19)
O1—Ti1—C4	98.28 (17)	C2A—C3A—C8A	125.2 (19)
O2—Ti1—C4	146.91 (14)	C4A—C3A—Ti1	73.9 (17)
O3—Ti1—C4	97.12 (18)	C2A—C3A—Ti1	76 (2)
C3A—Ti1—C4	24.9 (6)	C8A—C3A—Ti1	124 (3)
C4A—Ti1—C4	13.6 (5)	C3A—C4A—C5A	107.4 (11)
C2—Ti1—C4	57.41 (19)	C3A—C4A—C9A	122.2 (18)
O1—Ti1—C2A	97.6 (5)	C5A—C4A—C9A	130.3 (18)
O2—Ti1—C2A	97.6 (6)	C3A—C4A—Ti1	70.8 (19)
O3—Ti1—C2A	146.7 (6)	C5A—C4A—Ti1	74.9 (12)
C3A—Ti1—C2A	35.2 (6)	C9A—C4A—Ti1	121.5 (13)
C4A—Ti1—C2A	57.7 (7)	C1A—C5A—C4A	108.2 (12)
C2—Ti1—C2A	9.2 (5)	C1A—C5A—C10A	125.5 (19)
C4—Ti1—C2A	54.5 (6)	C4A—C5A—C10A	126.2 (19)
O1—Ti1—C1	139.7 (2)	C1A—C5A—Ti1	72.4 (17)
O2—Ti1—C1	90.41 (18)	C4A—C5A—Ti1	70.0 (12)
O3—Ti1—C1	110.0 (3)	C10A—C5A—Ti1	125.1 (16)
C3A—Ti1—C1	58.3 (7)	C1A—C6A—H6A1	109.5
C4A—Ti1—C1	48.8 (5)	C1A—C6A—H6A2	109.5
C2—Ti1—C1	34.5 (2)	H6A1—C6A—H6A2	109.5
C4—Ti1—C1	57.36 (18)	C1A—C6A—H6A3	109.5
C2A—Ti1—C1	42.5 (5)	H6A1—C6A—H6A3	109.5
O1—Ti1—C3	84.26 (19)	H6A2—C6A—H6A3	109.5
O2—Ti1—C3	123.8 (3)	C2A—C7A—H7A1	109.5
O3—Ti1—C3	131.0 (3)	C2A—C7A—H7A2	109.5
C3A—Ti1—C3	9.7 (5)	H7A1—C7A—H7A2	109.5
C4A—Ti1—C3	43.4 (5)	C2A—C7A—H7A3	109.5
C2—Ti1—C3	34.6 (2)	H7A1—C7A—H7A3	109.5
C4—Ti1—C3	34.26 (18)	H7A2—C7A—H7A3	109.5
C2A—Ti1—C3	27.1 (6)	C3A—C8A—H8A1	109.5
C1—Ti1—C3	57.3 (2)	C3A—C8A—H8A2	109.5
O1—Ti1—C1A	130.9 (5)	H8A1—C8A—H8A2	109.5
O2—Ti1—C1A	82.6 (5)	C3A—C8A—H8A3	109.5
O3—Ti1—C1A	122.6 (5)	H8A1—C8A—H8A3	109.5
C3A—Ti1—C1A	58.6 (8)	H8A2—C8A—H8A3	109.5
C4A—Ti1—C1A	58.4 (6)	C4A—C9A—H9A1	109.5
C2—Ti1—C1A	25.0 (5)	C4A—C9A—H9A2	109.5
C4—Ti1—C1A	64.3 (6)	H9A1—C9A—H9A2	109.5
C2A—Ti1—C1A	34.0 (5)	C4A—C9A—H9A3	109.5
C1—Ti1—C1A	13.8 (4)	H9A1—C9A—H9A3	109.5
C3—Ti1—C1A	54.9 (6)	H9A2—C9A—H9A3	109.5
O1—Ti1—C5A	143.9 (5)	C5A—C10A—H10D	109.5
O2—Ti1—C5A	104.6 (6)	C5A—C10A—H10E	109.5
O3—Ti1—C5A	92.2 (5)	H10D—C10A—H10E	109.5
C3A—Ti1—C5A	58.2 (8)	C5A—C10A—H10F	109.5
C4A—Ti1—C5A	35.2 (4)	H10D—C10A—H10F	109.5
C2—Ti1—C5A	50.5 (5)	H10E—C10A—H10F	109.5
C4—Ti1—C5A	47.2 (5)	O2—C11—C16	122.5 (3)
C2A—Ti1—C5A	56.4 (7)	O2—C11—C12	121.9 (3)
C1—Ti1—C5A	20.7 (5)	C16—C11—C12	115.6 (2)
C3—Ti1—C5A	61.3 (5)	F5—C12—C13	119.0 (3)
C1A—Ti1—C5A	34.3 (5)	F5—C12—C11	119.3 (3)
Ti1—O1—Ti1^i^	180.00 (4)	C13—C12—C11	121.7 (3)
C11—O2—Ti1	172.0 (2)	F4—C13—C14	119.4 (3)
C17—O3—Ti1	162.14 (19)	F4—C13—C12	120.0 (3)
C2—C1—C5	108.2 (4)	C14—C13—C12	120.6 (3)
C2—C1—C6	125.0 (9)	F3—C14—C15	120.3 (3)
C5—C1—C6	126.7 (8)	F3—C14—C13	120.1 (3)
C2—C1—Ti1	71.6 (4)	C15—C14—C13	119.6 (3)
C5—C1—Ti1	73.7 (3)	F2—C15—C14	120.1 (3)
C6—C1—Ti1	120.8 (5)	F2—C15—C16	119.5 (3)
C1—C2—C3	108.5 (5)	C14—C15—C16	120.3 (3)
C1—C2—C7	127.7 (9)	F1—C16—C15	118.8 (3)
C3—C2—C7	123.7 (9)	F1—C16—C11	119.1 (3)
C1—C2—Ti1	73.9 (4)	C15—C16—C11	122.2 (3)
C3—C2—Ti1	73.9 (5)	O3—C17—C22	122.4 (3)
C7—C2—Ti1	120.9 (5)	O3—C17—C18	121.9 (3)
C4—C3—C2	107.7 (5)	C22—C17—C18	115.7 (2)
C4—C3—C8	125.3 (8)	F6—C18—C19	118.4 (3)
C2—C3—C8	127.0 (9)	F6—C18—C17	119.6 (2)
C4—C3—Ti1	72.5 (4)	C19—C18—C17	122.0 (3)
C2—C3—Ti1	71.5 (5)	F7—C19—C20	119.6 (3)
C8—C3—Ti1	121.9 (6)	F7—C19—C18	120.2 (3)
C3—C4—C5	108.5 (5)	C20—C19—C18	120.2 (3)
C3—C4—C9	125.4 (7)	F8—C20—C21	120.2 (3)
C5—C4—C9	126.1 (7)	F8—C20—C19	120.1 (3)
C3—C4—Ti1	73.2 (5)	C21—C20—C19	119.7 (3)
C5—C4—Ti1	73.9 (3)	F9—C21—C20	119.8 (3)
C9—C4—Ti1	120.9 (3)	F9—C21—C22	120.4 (3)
C1—C5—C4	107.0 (4)	C20—C21—C22	119.8 (3)
C1—C5—C10	126.4 (7)	F10—C22—C21	118.7 (3)
C4—C5—C10	126.5 (7)	F10—C22—C17	118.6 (3)
C1—C5—Ti1	71.9 (3)	C21—C22—C17	122.7 (3)
C4—C5—Ti1	71.4 (3)		

Symmetry codes: (i) −*x*, −*y*, −*z*.
